# *Peribacillus castrilensis* sp. nov.: A Plant-Growth-Promoting and Biocontrol Species Isolated From a River Otter in Castril, Granada, Southern Spain

**DOI:** 10.3389/fpls.2022.896728

**Published:** 2022-06-23

**Authors:** Miguel Rodríguez, José Carlos Reina, Inmaculada Sampedro, Inmaculada Llamas, Fernando Martínez-Checa

**Affiliations:** ^1^Department of Microbiology, Faculty of Pharmacy, University of Granada, Granada, Spain; ^2^Biomedical Research Centre (CIBM), Institute of Biotechnology, University of Granada, Granada, Spain

**Keywords:** *Peribacillus castrilensis*, river otter, new species, plant-growth promoting species, phytopathogen, quorum quenching

## Abstract

A strictly aerobic, chemoheterotrophic, endospore-forming, Gram-positive, rod-shaped bacterial strain N3^T^ was isolated from the feces of a river otter in Castril (Granada, southern Spain). It is halotolerant, motile, and catalase-, oxidase-, ACC deaminase-, and C4- and C8-lipase-positive. It promotes tomato plant growth and can reduce virulence in *Erwinia amylovora* CECT 222^T^ and *Dickeya solani* LMG 25993^T^ through interference in their quorum-sensing systems, although other antagonistic mechanisms could also occur. A phylogenetic analysis of the 16S rRNA gene sequence as well as the phenotypic and phylogenomic analyses indicated that the strain N3^T^ is a novel species of the genus *Peribacillus*, with the highest 16S rRNA sequence similar to that of *Bacillus frigoritolerans* DSM 8801^T^ (99.93%) and *Peribacillus simplex* DSM 1321^T^ (99.80%). Genomic digital DNA–DNA hybridization (dDDH) between the strain N3^T^ and *Bacillus frigoritolerans* DSM 8801^T^ and *Peribacillus simplex* was 12.8 and 69.1%, respectively, and the average nucleotide identity (ANIb) of strain N3^T^ and *Bacillus frigoritolerans* DSM 8801^T^ and *Peribacillus simplex* was 67.84 and 93.21%, respectively. The genomic G + C content was 40.3 mol%. Its main cellular fatty acids were anteiso-C_15:0_ and iso-C_15:0_. Using 16S rRNA phylogenetic and *in silico* phylogenomic analyses, together with the chemotaxonomic and phenotypic data, we demonstrated that the type strain N3^T^ (=CECT 30509^T^ = LMG 32505^T^) is a novel species of the genus *Peribacillus* and the name *Peribacillus castrilensis* sp. nov. is proposed.

## Introduction

Considerable economic losses in agriculture are caused every year in a wide range of crops worldwide by the diseases caused by the plant bacterial pathogens (Kannan et al., 2015; Martins et al., 2018). To combat these infections, chemical pesticides and antibiotics have been used for many decades (Aktar et al., 2009; Manyi-Loh et al., 2018), causing serious problems such as soil salinization, environmental pollution, and a reduction in productivity due to resistance to treatment. Antibiotic resistance constitutes a serious risk to the progress made by the global health and international organizations, such as WHO and FAO, which are focused on the search for alternative bacterial control strategies to combat plant diseases and to promote plant growth with more sustainable eco-friendly approaches (FAO, 2017).

Currently, one of the most promising alternatives in the agricultural industry is the use of formulations containing plant-growth-promoting bacteria (PGPB) and other beneficial microorganisms, that are used as biofertilizers, which can interact with plant pathogens (Vessey, 2003; Borriss, 2011; Khatoon et al., 2020). They inhibit the pathogen growth through the synthesis of antibiotics, bacteriocins, and hydrolytic enzymes, the competition for nutrients and parasitism, and the physical displacement of these pathogens (Borriss, 2011; Kumari et al., 2019).

Another promising strategy to control agricultural bacterial diseases is the interference of quorum-sensing (QS) systems in plant pathogens. QS is an intercellular communication system in which the expression of some bacterial genes is mediated by specific signal molecules known as autoinducers. They are produced, diffused to the external medium, and recognized by other bacteria in a cell-density-dependent manner. Among these, *N*-acylhomoserine lactones (AHLs) are the most common and are produced by many *Pseudomonadota* (Fuqua et al., 1994). Different bacterial phenotypes are regulated by this system, many of which have been shown to contribute to bacterial virulence in a number of economically important agriculture pathogens (Von Bodman et al., 2003). For this reason, the interruption of QS is an interesting strategy against bacterial infections in plants (Grandclément et al., 2015). In this sense, one of the best described QS-interrupting strategies is known as quorum quenching (QQ) based on the enzymatic degradation of the AHL signal molecules (Uroz et al., 2009). These signal molecules can be degraded or even modified by different enzymes, including acylases, lactonases, and oxidoreductases (Fetzner, 2015). Through this strategy, it has been already demonstrated that the reduction of the virulence of several plant bacterial pathogens produces promising results (Uroz et al., 2003; Faure and Dessaux, 2007; Helman and Chernin, 2015).

During the course of a study of feces of river otters living in Castril, Granada, southern Spain, the strain N3^T^ was isolated in pure culture. On the basis of a polyphasic analysis, we demonstrated that it is a new species within the genus *Peribacillus*. It is known that river otters usually contain the members of Phylum *Firmicutes* as the dominant group in their intestinal microbiota (An et al., 2017).

The species of the genus *Peribacillus* were originally included in the genus *Bacillus*. The genus *Bacillus*, first described in 1872 by Logan and De Vos (2009), comprises 102 species and subspecies. It has been recently reclassified into new genera, *Neobacillus, Mesobacillus, Metabacillus, Cytobacillus, Alkalihalobacillus, Peribacillus*, and more; only the subtilis and cereus clades are left in the genus *Bacillus* (Gupta et al., 2020; Patel and Gupta, 2020).

At the time of writing, the genus *Peribacillus* comprises 17 species with validly published names (https://lpsn.dsmz.de/genus/peribacillus); most of them have been isolated from a variety of ecological niches, including soil (Yumoto et al., 2004; Lim et al., 2007; Kuisiene et al., 2008; Zhang et al., 2011; Li et al., 2014; Feng et al., 2016; Liu et al., 2016; Ma et al., 2018), plant tissues (Zhang et al., 2012; Kämpfer et al., 2015), and cow feces (Jiang et al., 2019). The species of this genus are Gram-positive, the cells are motile, aerobic, or facultatively anaerobic, and the growth occurs at the temperature range between 3 and 45°C. The whole-genome sequences are available only for 11 species with validly published names and range in sizes from 4.1 to 5.7 Mbp. The genomic DNA G+C contents range from 37.5 to 43.0 mol%.

Using a polyphasic taxonomic approach, and based on the differences in the phenotypic, chemotaxonomic, and genetic distinctiveness (ANI and dDDH), the strain N3^T^ should be recognized as a novel species of the genus *Peribacillus*, for which we propose the name of *Peribacillus castrilensis*. We set out, therefore, to analyze the potential of the *Peribacillus* sp. strain N3^T^ as a plant-growth-promoting and biocontrol agent against phytopathogens.

## Materials and Methods

### Bacterial Strains, Compounds, Media, and Growth Conditions

The strain N3^T^ was isolated from the feces of a river otter in Castril, Granada, southern Spain (37°52′00″N 2°45′58″W). One gram of feces was suspended in 0.9% (w/v) saline solution to a final volume of 10 mL. A volume of 0.1 mL of the sample was then plated on a tryptone soy agar (TSA) medium and incubated at 28°C for 7 days. The different isolated colonies were subsequently plated and purified on the same medium.

The strain N3^T^ and the phytopathogenic strains *Erwinia amylovora* CECT 222^T^ and *Dickeya solani* LMG 25993^T^ were grown in a tryptic soy broth (TSB) medium. *Agrobacterium tumefaciens* NTL4 (pZLR4) was grown in a Luria Bertani (LB) medium supplemented with 2.5 mmol L^−1^ CaCl_2_ 2H_2_O,2.5 mmol L^−1^ MgSO_4_ 7H_2_O (LB/MC), and gentamicin (Gm) to a final concentration of 50 μg mL^−1^. *Chromobacterium violaceum* CV026 and *C. violaceum* VIR07 were grown in an LB medium supplemented with 50 μg mL^−1^ kanamycin (Km). All the strains were grown at 28°C at 100 rpm in a rotary shaker.

The synthetic AHLs (Sigma-Aldrich, Saint Louis, USA) used were as follows: C4-HSL (N-butyryl-DL-homoserine lactone), C6-HSL (N-hexanoyl-DL-homoserine lactone), 3-O-C6-HSL (N-3-oxo-hexanoyl-DL-homoserine lactone), C8-HSL (N-octanoyl- DL -homoserine lactone), 3-O-C8-HSL (N-3-oxo-octanoyl-DL-homoserine lactone), C10-HSL (N-decanoyl-DL-homoserine lactone), 3-OH-C10-HSL (N-3-hydroxydecanoyl-DL-homoserine lactone), C12-HSL (N-dodecanoyl-DL-homoserine lactone), and 3-O-C12-HSL (N-3-oxo-dodecanoyl-DL-homoserine lactone).

### Phylogenetic 16S rRNA Gene Analysis

The genomic DNA was isolated by using the X-DNA Purification Kit (Xtrem Biotech S.L., Granada, Spain), and the 16S rRNA gene was amplified by the universal bacterial primers 16F27 and 16R1488. The PCR product was purified and cloned into the pGEM®-T vector (Promega). Direct sequencing of the PCR-amplified DNA was determined using an ABI PRISM DyeTerminator Cycle Sequencing Ready Reaction Kit (Perkin-Elmer) and an ABI PRISM 377 Sequencer (Perkin-Elmer) according to the manufacturer's instructions. The DNA sequence obtained was compared to the reference 16S rRNA gene sequences available in the GenBank and EMBL databases obtained from the NCBI Genome Database using BLASTN software (Altschul et al., 1990) and the EzBioCloud server (Yoon et al., 2017). The phylogenetic analysis was carried out using Molecular Evolutionary Genetics Analysis (MEGA) software version X (Kumar et al., 2018) following multiple data alignments by the CLUSTAL OMEGA (Sievers et al., 2011). Distances and clustering were determined according to the neighbor-joining and maximum-likelihood methods by applying the cluster stability algorithm based on the bootstrap analysis (1,000 replications).

### Phenotypic and Chemotaxonomic Characterization

To describe new taxa of aerobic and endospore-forming bacteria, the recommended traits by Logan et al. (2009) were applied in the study of the type species *Peribacillus*. The shape, size, and pigmentation of the colonies were observed on a TSA medium after 48 h of incubation at 28°C. The motility was observed using a log phase culture according to the Hanging drop method. The oxidase (Kovacs, 1956) and catalase activities were determined as well.

The optimum growth and growth range were determined in a TSB medium at different NaCl concentrations ranging from 0 to 25% (w/v) in 1.0 intervals adjusting the pH 7. The pH growth range and optimum pH were also determined in the TSB medium, testing from 4 to 11 in 1.0 pH unit intervals, using the following buffer systems: 0.1 M citric acid/0.1 M sodium citrate (pH 4.0–5.0,); 0.1 M KH_2_PO_4_/0.1 M NaOH (pH 6.0–8.0); 0.1 M NaHCO_3_/0.1 M Na_2_CO_3_ (pH 9.0–10.0); and 0.2 M KH_2_PO_4_/0.1 M NaOH (pH 11.0) (Xie et al., 2012). In both tests, bacterial growth was monitored by optical density at 600 nm. The temperature range for growth and the optimum temperature were determined on the TSA plates at 4, 10, 15, 20, 28, 37, 40, 42, and 45°C. The anaerobic growth capacity was evaluated on the TSA plates by incubation in hermetic jars using the Gas Pak Anaerobic System (BBL) to generate an anaerobic atmosphere over a one-week period. The hydrolyses of casein and starch were also performed (Uttley and Collins, 1993). Other biochemical characteristics were analyzed using the API 50CH and API 20E according to the manufacturer's instructions.

The cellular fatty acids were analyzed at the Spanish Type Culture Collection (CECT) in Valencia, Spain, following the instructions of the Microbial Identification System Operating Manual (MIDI, 2008). For this, the cell mass of the N3^T^ strain was obtained after growing for 24 h in a TSB medium at 28°C.

### Genome Sequencing and Assembly

The genomic N3^T^ strain DNA was extracted following the protocol described by Marmur (1961) for later sequencing by the Illumina Hi-Seq platform at the STAB VIDA facility (Caparica, Portugal) with 2 × 150-bp paired-end reads. The reads that were processed by BBDuk (https://sourceforge.net/projects/bbmap/) to remove the adapters and low-quality bases were then assembled using SPAdes software v. 3.11.1 (Nurk et al., 2013). Finally, the obtained contigs were blasted against the nr/nt database to remove the contigs belonging to the contaminants.

### *In silico* ANI, AAI, and DDH

The average nucleotide identity based on BLAST (ANIb) and MUMmer (ANIm) algorithms were determined with the aid of JSpeciesWS software (Richter et al., 2015). OrthoANI was similarly calculated using OrthoANI software (Lee et al., 2016). The average amino acid identity (AAI) values were calculated from protein sequences using an online AAI calculator at the Kostas Laboratory website (http://enve-omics.ce.gatech.edu/aai/). A two-way AAI was used.

In the case of digital DNA–DNA hybridization (dDDH), it was calculated using the BLAST+ algorithm on the DSMZ Genome-to-Genome Distance Calculator (GGDC 3.0) platform (Meier-Kolthoff et al., 2013, 2021). The results presented in this study are based on the recommended Formula 2 (identities/HSP length), which, being independent of genome length, is robustly protected against the use of incomplete draft genomes.

### Analysis of the Core Orthologous Genes

A core genome analysis of the strain N3^T^ and all species of the genus *Peribacillus*, including a representative strain of the related genera, for which their genome was available, was also performed using Bacterial Pan Genome Analysis (BPGA) software (Chaudhari et al., 2016) with the default parameters. After obtaining the core of the 23 bacterial genomes, all protein orthologs belonging to the core genome were concatenated and aligned by MAFFT (Katoh and Standley, 2013). A phylogenomic tree of the core genes of the species was then constructed using MEGA X software according to the maximum-likelihood method.

### Determination of the Possible Mechanisms of N3^T^ Action

*In vitro* plant-growth-promoting (PGP) traits were analyzed for the N3^T^ strain through the screening of the production of acid and alkaline phosphatases (Pikovskaya, 1948; Baird-Parker, 1963), 1-aminocyclopropane-1-carboxylic acid (ACC) deaminase (Poonguzhali et al., 2006), indoleacetic acid (IAA) (Gang et al., 2019), and siderophores (Alexander and Zuberer, 1991) and the ability to fix nitrogen (Matthews and Suhaimi, 2010).

Moreover, rhizosphere competence traits were studied in this strain by the production of enzymes related to the hydrolyses of casein, cellulose, DNA, gelatin, starch, Tween 20, and Tween 80 (Jeffries et al., 1957; Uttley and Collins, 1993; Villalba et al., 2004). In the case of casein, cellulose, DNA, starch, and acid phosphatase, a clear halo surrounding the bacterial growth indicated a positive result for these tests, while the precipitation of calcium salts is detected in the case of Tween 20 and 80 hydrolyses. Alkaline phosphatase production is detected by a pink coloration after adding 10 mL of 30% (v/v) of ammonia to the plate. In the case of nitrogen fixation and ACC deaminase production, the growth of bacterial strain indicated a positive result by fixing gaseous nitrogen or degrading ACC, as the media do not contain any source of N except for ACC. The Siderophore test was carried out by the chrome azurol sulfonate (CAS) protocol, in which a change of color from blue to green was recorded as a positive result. Meanwhile, IAA production was detected spectrophotometrically in a TSB medium supplemented with tryptophan (500 mg/L) after the addition of the Salkowski reagent.

The N3^T^ strain QQ activity was assessed by a well-diffusion agar-plate assay using synthetic AHLs (Romero et al., 2011; Torres et al., 2016). Briefly, 10 μM of each AHL was added to an overnight culture of the N3^T^ strain and then incubated at 28°C for 24 h. A sterile TSB medium supplemented with AHLs was incubated as a negative control. The remaining AHLs were detected in the supernatant of each sample that was deposited in wells on the LB agar plates overlaid with *C. violaceum* CV026 or *C. violaceum* VIR07 or on AB agar plates supplemented with 80 μg mL^−1^ of 5-bromo-4-chloro-3-indolyl-ß-D-galactopyranoside (Xgal) overlaid with *Agrobacterium tumefaciens* NTL4 (pZLR4). The plates were incubated at 28°C for 24 h to check for the development of a purple or blue color around each well. This assay was repeated three times.

### Determination of Plant-Growth-Promoting and Biocontrol Activities

For plant growth promotion assay in tomato plants, 50 seeds were surface sterilized (Molan et al., 2010) and sown in each 20 × 20 × 20 cm pot containing sterile vermiculite. When seedlings were 5 cm, each pot was irrigated with 5 mL of 10^9^ CFU mL^−1^ of the strain N3^T^-washed cells every seven days. Irrigation with sterile distilled water was used as a negative control. Pots were kept in a greenhouse under a long-day photoperiod (16:8h, light:dark) at 21°C for 4 weeks. Then, root and shoot lengths and dry weight were determined.

To test the ability of the strain N3^T^ to interfere in the *D. solani* LMG 25993^T^ and *E. amylovora* CECT 222^T^ virulence, experiments using cocultures between them were carried out in potato slices and pears, respectively. Briefly, pathogen (10^7^ CFU mL^−1^)–strain N3^T^ (10^9^ CFU mL^−1^) cocultures were conducted in a 1:100 ratio in a TSB medium and incubated for 24 h at 28°C. A similar concentration of each pathogen or strain N3^T^ was added to the cell-free TSB as controls, respectively (Torres et al., 2016). For the potato tubers (*Solanum tuberosum*) and pears (*Pyrus communis*) assays, they were tap-washed and surface-sterilized by spraying with 1% (w/v) sodium hypochlorite solution, followed by 70% (v/v) ethanol and sterile distilled water. Sliced potatoes and pears were inoculated with 5 μL of the N3^T^-phytopathogen cocultures and with controls (each bacterium monocultures and the sterile distilled water) at three or four equidistant spots, respectively. Nine replicates of each treatment were performed, and the experiment was repeated three times. After 48 h of incubation at 28°C, the maceration zones were visually detected and measured using ImageJ software (Schneider et al., 2012). Plate counts in monocultures and cocultures were performed to determine the concentration of each phytopathogen and strain N3^T^ in the TSA medium supplemented with 5% (w/v) NaCl as a selective agent for excluding the salt-sensitive phytopathogens.

In parallel, the remaining AHLs from each culture were determined according to the well-diffusion agar-plate method to assess the QQ activity of the strain N3^T^. For this, the co-cultures were centrifuged at 12,000 rpm for 10 min, and the cell-free supernatants containing the AHLs were subject to a double extraction with an equal volume of dichloromethane. The organic phase was dried and finally suspended in 20 μL of 70% (v/v) methanol. Five microliters of each extract were spotted in the sterile filter paper disks placed in AB-Xgal plates (Chilton et al., 1974) using *A. tumefaciens* NTL4 (pZLR4) as a biosensor. The monocultures of the strain N3^T^ and each pathogen were likewise extracted and tested as negative and positive controls, respectively.

### Statistical Analysis

The Shapiro–Wilk test was used to verify data normality, and the data were statistically analyzed with the aid of the ANOVA (*P* ≤ 0.05) and Tukey tests using the SPSS software.

## Results and Discussion

### Phylogenetic Analysis Based on the 16S rRNA Gene Sequence

**Figure 1:**
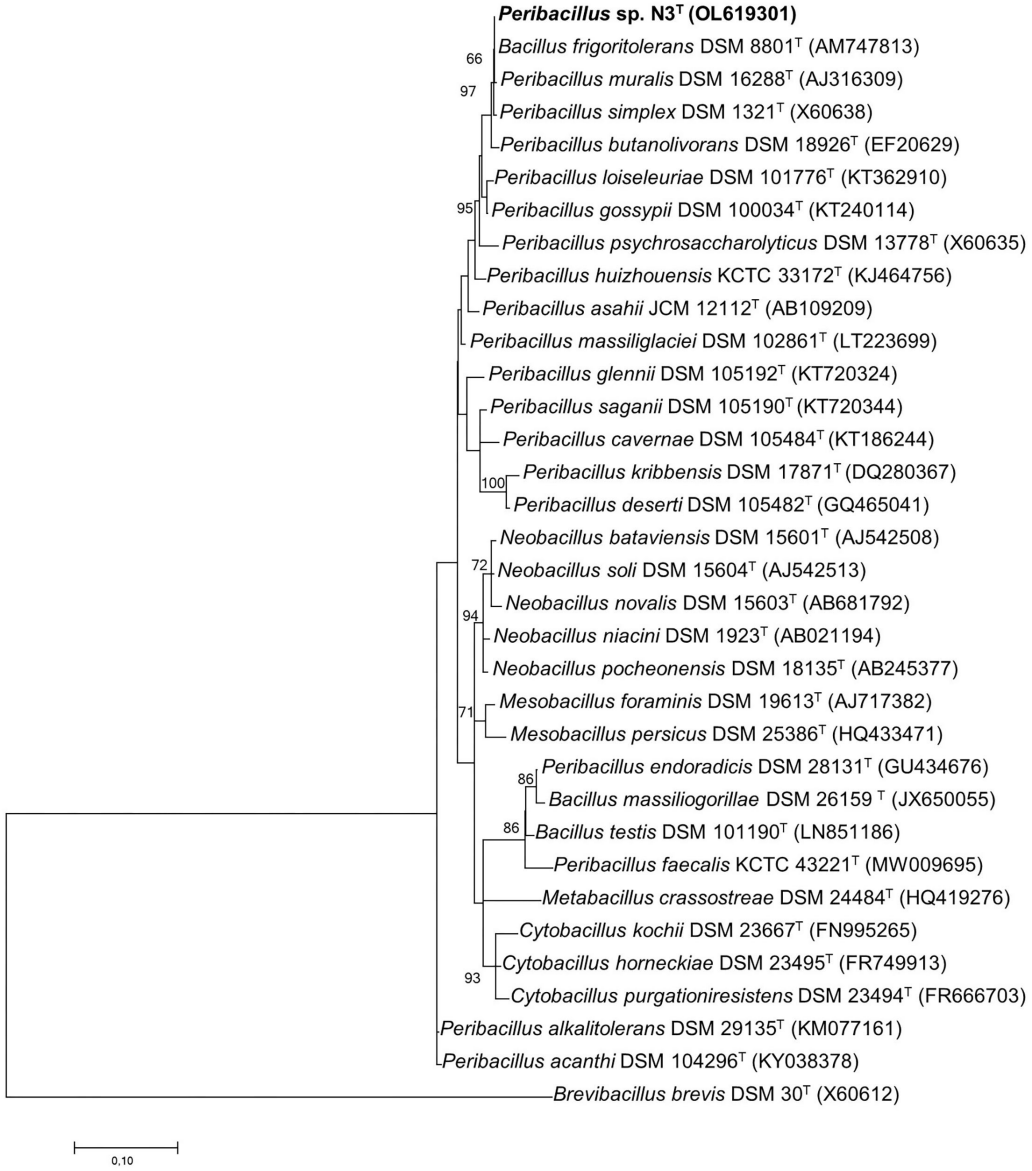
Phylogenetic position of the strain N3^T^ the 16S rRNA gene sequence (bold) and its relationship with other related species by using the maximum-likelihood algorithm based on the Kimura 2-parameter model. A discrete Gamma distribution was used to model evolutionary rate differences among sites [five categories (+G, parameter = 0.2703)]. The GenBank/EMBL/DDBJ accession number of each sequence is shown in parenthesis. Bootstrap values are expressed as percentages of 1,000 replications, and those >60% are shown at branch points. Bar shows sequence divergence. Bar−0.01 substitutions per nucleotide position. *B. brevis* DSM 30^T^ sequence was used as an outgroup.

The cloned 16S rRNA gene of the strain N3^T^ resulted in a virtually complete 1558 bp-long sequence. The strain N3 ^T^ showed the highest sequence identity to *Bacillus frigoritolerans* DSM 8801^T^ (99.93%), *Peribacillus simplex* DSM 1321^T^ (99.80%), *P. muralis* DSM 16288^T^ (99.66%), *P. butanolivorans* DSM 18926^T^ (99.59%), and *P. loiseleuriae* (97.96%), while identities below 97% were obtained with other species from *Peribacillus* genus. Further phylogenetic analysis of its 16S rRNA gene sequences and other related strains through a phylogenetic tree reconstruction using the maximum-likelihood algorithm showed that this strain is a member of the genus *Peribacillus* and forms a cluster with the *B. frigoritolerans* species, which showed the highest sequence similarity (Figure 1). A similar phylogenetic distribution was achieved when neighbor-joining and maximum parsimony algorithms were applied (Supplementary Figures 1, 2, respectively).

### Phenotypic and Chemotaxonomic Characterization

The strain N3^T^ forms creamy and convex colonies of 4–5 mm diameter after 48 h of incubation in the TSA plates. The cells were rod-shaped, sometimes chained and motile when single cells, Gram-positive, catalase-positive, and oxidase-negative. The strain N3^T^ produced oval endospores in swollen sporangia. The strain N3^T^ was strictly aerobe, with growth temperatures ranging from 10 to 42°C and with an optimum temperature at 28°C. It grew in a pH range from 6 to 8, with pH 7 as optimum. This strain proved to be halotolerant, as it grew from 0.5 to 7.5% (w/v) of NaCl, with 1% (w/v) being the optimum concentration.

Its phenotypic characteristics are shown in Table 1 and in the species description section. The differential characteristics of the strain N3^T^ with respect to the most closely related species, *B. frigoritolerans* DSM 8801^T^*, P. muralis* DSM 16288^T^, *P. butanolivorans* DSM 18926^T^, *P. loiseleuriae* DSM 101776^T^, and the type strain of the genus *P. simplex* DSM 1321^T^ are also shown in Table 1. The strain N3^T^ mainly differs in relation to the following features: the inability of growing anaerobically and at 4 or 45°C, hydrolyze casein, reducing nitrate to nitrite, and resulting negative for oxidase, ONPG reaction, citrate utilization, and urease production, and none of the features were able to produce acids from any sugar tested in API 50CH except for aesculin, in which a weak result was detected. By contrast, this strain can grow in a wider range of NaCl and hydrolyze starch and gelatin and is also positive for arginine dihydrolase, tryptophan deaminase, and indole production.

**Table 1:** Differential characteristics between the N3^T^ strain with respect to its closest relative species.

**Characteristic**	**1**	**2**	**3**	**4**	**5**	**6**
Anaerobic growth	-	-	v	w	-	-
Growth at 4°C	-	+	-	-	+	-
Growth at 45°C	-	-	-	-	+	-
NaCl range (%) (w/v)	0.5–7.5	0.5–7.5	<5	<7	0.5–5	0–3
NaCl optimum (%) (w/v)	1	0	0	0	1	0
pH range	6–8	5–10	6–9	6–9	6–9	6–9
pH optimum	7	7	8	7	7	7
Oxidase	-	-	-	+	+	-
Hydrolysis of:						
Starch	+	-	+	+	-	+
Casein	-	+	v	v	-	-
Nitrate reduction	-	v	+	+	+	+
ONPG	-	-	-	+	-	-
Arginine dihydrolase	+	-	-	-	-	-
Citrate utilization	-	-	-	-	-	+
Urease	-	-	-	-	-	+
Tryptophan deaminase	+	-	-	-	-	-
Indole production	+	-	-	-	-	+
Gelatine hydrolysis	+	-	v	-	-	-
Acids from carbohydrates:						
Aesculin	w	-	w/v	+	w/v	+
Arbutin	-	-	-	+	-	+
Cellobiose	-	+	-	+	-	+
Erytritol	-	-	-	-	-	+
Fructose	-	+	w	+	-	+
Galactose	-	-	-	+	-	-
Glucose	-	+	w	+	-	+
Glycerol	-	-	-	+	-	-
Inositol	-	+	-	-	-	+
Inulin	-	-	w	-	w	-
Lactose	-	-	-	+	-	+
L-arabinose	-	+	-	+	-	-
Maltose	-	-	-	+	-	+
Mannitol	-	-	-	+	-	+
Mannose	-	+	-	+	-	-
Melibiose	-	-	-	+	-	+
N-acetylglucosamine	-	-	w	+	w	-
Raffinose	-	+	-	+	-	+
Ribose	-	-	-	+	-	+
Salicin	-	-	w/v	+	w/v	-
Sucrose	-	+	w	v	-	+
Trehalose	-	-	w	+	w	+
DNA G + C content (mol%)	40.3	40.6	39.9	41.2	37.4	37.5

*Strains: 1, Peribacillus sp. N3^T^; 2, B. frigoritolerans DSM 8801^T^ (data from Liu et al., 2020); 3, P. simplex DSM 1321^T^ (data from Heyrman et al., 2005); 4, P. muralis DSM 16288^T^ (data from Heyrman et al., 2005); 5, P. butanolivorans DSM 18926^T^ (data from Kuisiene et al., 2008); 6, P. loiseleuriae DSM 101776^T^ (data from Liu et al., 2016). +, positive; -, negative; v, variable; w, weak; w/v, weak and variable*.

*All the strains were negative for lysine decarboxylase, ornithine decarboxylase, and H_2_S production and were not able to produce acids from D-xylose, L-xylose, adonitol, β-methyl-D-xyloside, sorbose, rhamnose, dulcitol, sorbitol, 1-methyl-D-mannoside, amygdaline, melezitose, starch, glycogen, gentiobiose, D-turanose, D-lyxose, D-tagatose, D-fucose, L-fucose, D-arabitol, L-arabitol, gluconate, 2-keto-gluconate, and 5-keto-gluconate*.

The analysis of the strain N3^T^ fatty acids indicated a predominance of anteiso-C_15:0_ (68.07%) and iso-C_15:0_ (8.71%) (Table 2). This profile was similar to that of the most closely related strains and the type strain of the genus; however, a marked increase in the relative abundance of anteiso-C_15:0_ and a decrease of iso-C_15:0_ made a difference with respect to its relative profiles.

**Table 2:** Cellular fatty acid content of N3^T^ and related species of the *Peribacillus* genus.

**Cellular fatty acids**	**1**	**2**	**3**	**4**	**5**	**6**
Saturated fatty acids:						
C_14:0_	1.22	1.5	1.56	4.35	1.76	6.10
C_15:0_	ND	ND	ND	1.21	1.33	ND
C_16:0_	3.47	3.8	2.36	2.27	2.97	6.80
Branched-chain fatty acids:						
iso-c_14:0_	3.04	8.3	5.94	8.67	8.77	8.60
iso-c_15:0_	8.71	23.6	15.55	22.51	16.78	17.90
anteiso-c_15:0_	68.07	48.8	59.03	42.69	45.80	53.20
iso-c_16:0_	2.68	2.0	2.26	1.61	4.48	ND
iso-c_17:0_	1.12	ND	ND	ND	1.27	ND
anteiso-c_17:0_	3.40	1.2	1.82	ND	2.72	ND
Unsaturated fatty acids:						
C_16:1_ ω7*c* alcohol	2.32	3.6	2.97	4.11	5.79	ND
C_16:1_ ω11*c*	4.12	2.7	4.8	10.33	6.14	2.20
iso-C_17:1_	1.85	ND	1.12	ND	ND	ND
Summed feature:						
C_17:1_ iso I/anteiso B	1.85	ND	1.12	ND	ND	ND

*Strains: 1, Peribacillus sp. N3^T^; 2, B. frigoritolerans DSM 8801^T^ (data from Liu et al., 2020); 3, P. simplex DSM 1321^T^ (data from Heyrman et al., 2005); 4, P. muralis DSM 16288^T^ (data from Heyrman et al., 2005); 5, P. butanolivorans DSM 18926^T^ (data from Kuisiene et al., 2008); and 6, P. loiseleuriae DSM 101776^T^ (data from Liu et al., 2016). ND, not detected or lower than 1% of the total composition*.

### Whole-Genome Sequencing and Assembly

The draft genome of strain N3 ^T^ was manually curated, and it resulted in over 5.7 Mbp with 88 contigs. The quality of the assembly was assessed using Quality Assessment Tool for Genome Assemblies (QUAST) software. The whole-genome sequence was of sufficient quality, with an N50 value of 263,554, an L50 value of 9, and approximately 200X coverage. The PGAP (Tatusova et al., 2016) annotation of the draft genome showed a total of 5,319 protein-coding genes (PCGs), 4,825 of which were assigned at least to a single functional COG category in the EggNOG 5.0 database (Huerta-Cepas et al., 2018; Cantalapiedra et al., 2021); categories K (transcription) and E (amino acid metabolism and transport) were the most abundant, with 455 and 585 proteins assigned to those categories, respectively. This genome sequence, which was deposited in the GenBank/EMBL/DDBJ database under accession number JAJNAF000000000, was used for further analysis.

### *In silico* G+C Content, ANI, AAI, and DDDH Calculations

The *in silico* analysis of G+C content in the draft genome of the strain N3^T^ produced a value of 40.28 mol%, whereas the range of G+C content for the type species of the genus *P. simplex* DSM 1321^T^ is 39.5–41.6 mol% (Heyrman et al., 2005).

The ANIs, based both on BLAST (ANIb) and MUMmer (ANIm), and the average AAI for the strain N3^T^ and the related species are shown in Supplementary Table 1. The ANIb and ANIm values between the strain N3^T^ and the most closely related *P. simplex* DSM 1321^T^ were 93.21% and 93.94%, respectively. The proposed cutoff for species delimitation is 95–96%, as proposed by Richter and Rosselló-Móra (Richter and Rossello-Mora, 2009; Kim et al., 2014). In all the cases, the ANI values for the strain N3^T^ and all the related species were below that cutoff. The AAI values between strain N3 and phylogenetically related species ranged from 67.54 to 95.67% (Supplementary Table 1). Konstantinidis and Tiedje (2005) proposed an AAI threshold (about 95–96%) for species demarcation of prokaryotes based on 175 genomes. The low AAI values confirm that the strain N3^T^ represents a novel species within the genus *Peribacillus*.

The OrthoANI calculation between the N3^T^ strain and *P. simplex* DSM 1321^T^ was 93.66%, which is in line with the results obtained for ANIb and ANIm and below the proposed cutoff (Supplementary Table 2).

The dDDH of the whole-genome sequences of the N3^T^ strain and the closely related species was carried out, and in all the cases, the results fell below the proposed cutoff delimitation for the species (70%) (Goris et al., 2007) (Supplementary Table 2).

The data obtained with *Bacillus frigoritolerans* (Liu et al., 2020) also indicated that this taxon must be reclassified as *Peribacillus frigoritolerans*.

### Phylogenetic Analysis of Core Orthologous Proteins

**Figure 2:**
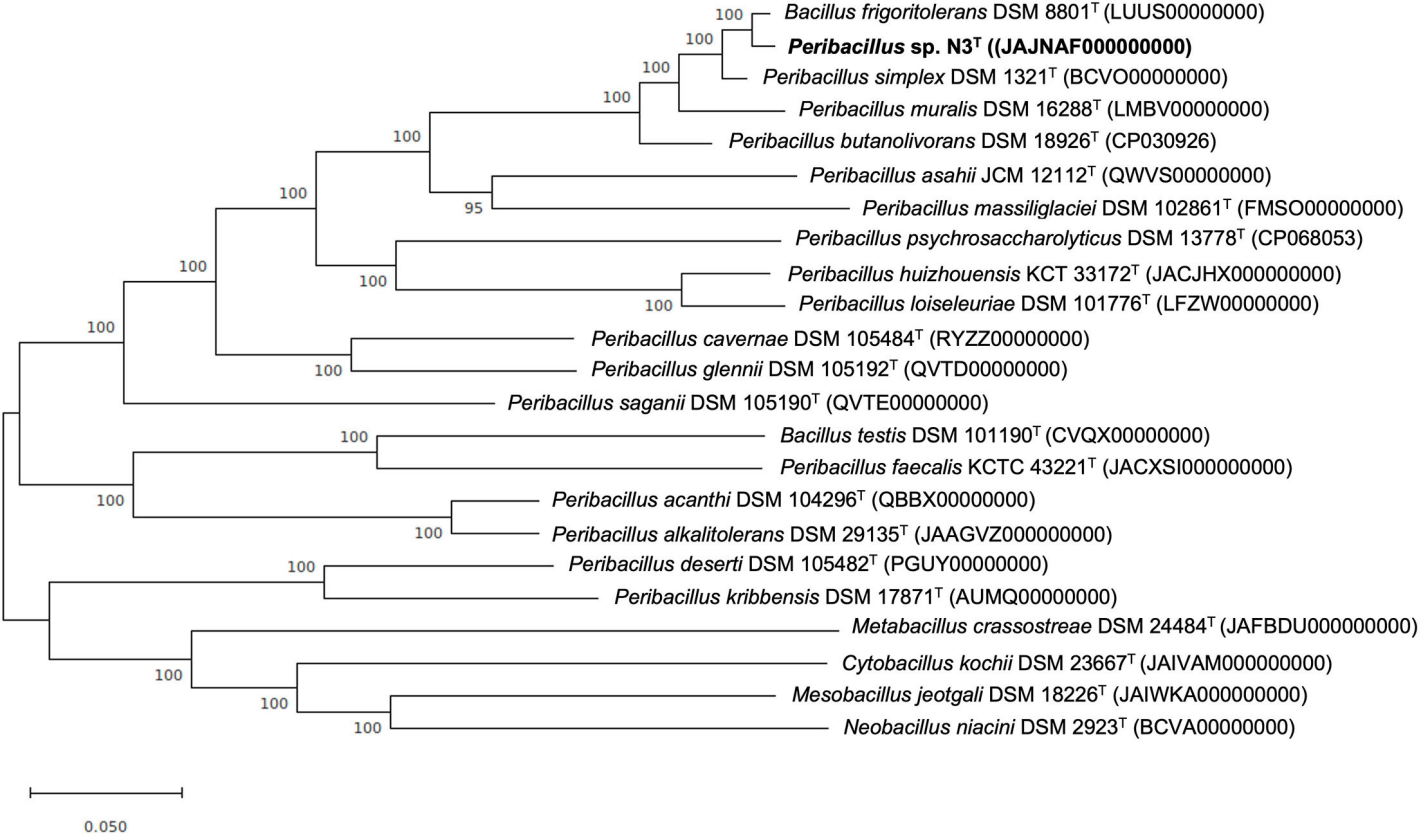
Tree constructed according to the maximum-likelihood method based on 803 core orthologous proteins of the strain N3^T^ (bold) and the available genomes of *Peribacillus* species and representative strains of the related genera. Bootstrap values are expressed as percentages of 1,000 replications, and those over 60% are shown at branch points. Bar−0.05 substitutions per nucleotide position.

The concatenated alignment of the 803 core orthologous proteins of the strain N3^T^ and the species of the genus *Peribacillus*, including a representative strain of the related genera, was used to reconstruct a maximum-likelihood phylogenetic tree, confirming our previous results, as shown in Figure 2.

### Biochemical Characterization of Plant Growth Promotion Activity and Quorum Quenching Traits

The strain N3^T^ resulted positive for DNA, gelatin, starch, and Tween 20 and Tween 80 hydrolyses. It also produced siderophores and IAA and was able to fix nitrogen and degrade ACC, while the acid phosphatase activity was variable (Supplementary Table 3). Some of the hydrolyses are associated with plant nutrient acquisition, pathogen competition, and phytohormone balance interference in the plant with the increase of plant growth and development hormones and the decrease of stress hormones (Gupta and Pandey, 2019; Miljaković et al., 2020).

**Figure 3:**
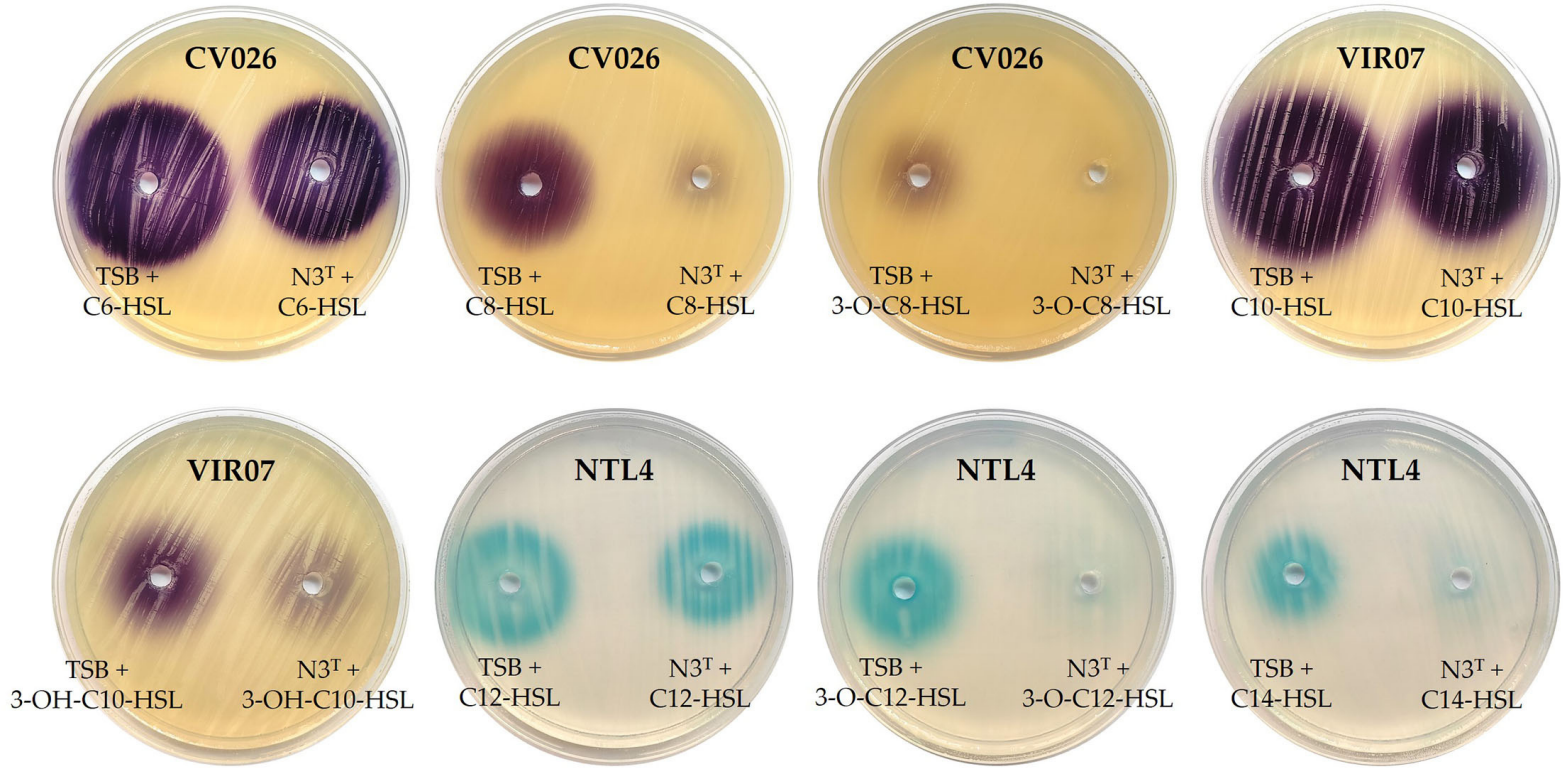
Determination of N3^T^ quorum quenching activity on synthetic AHLs. *C. violaceum* CV026, *C. violaceum* VIR07, and *A. tumefaciens* NTL4 (pZLR4) were used as biosensors in LB and AB-Xgal media, respectively.

Concerning the QQ activity of this strain, it was able to degrade the majority of the synthetic AHLs tested with different intensities depending on the AHL. The highest degradation was achieved against 3-O-C6-HSL and 3-O-C12-HSL, in which a total degradation was observed. By contrast, a lower degradation was detected for C6-HSL, C10-HSL, and C12-HSL (Figure 3).

### Growth Promotion in Tomato Plants and Biocontrol of Phytopathogens

**Figure 4:**
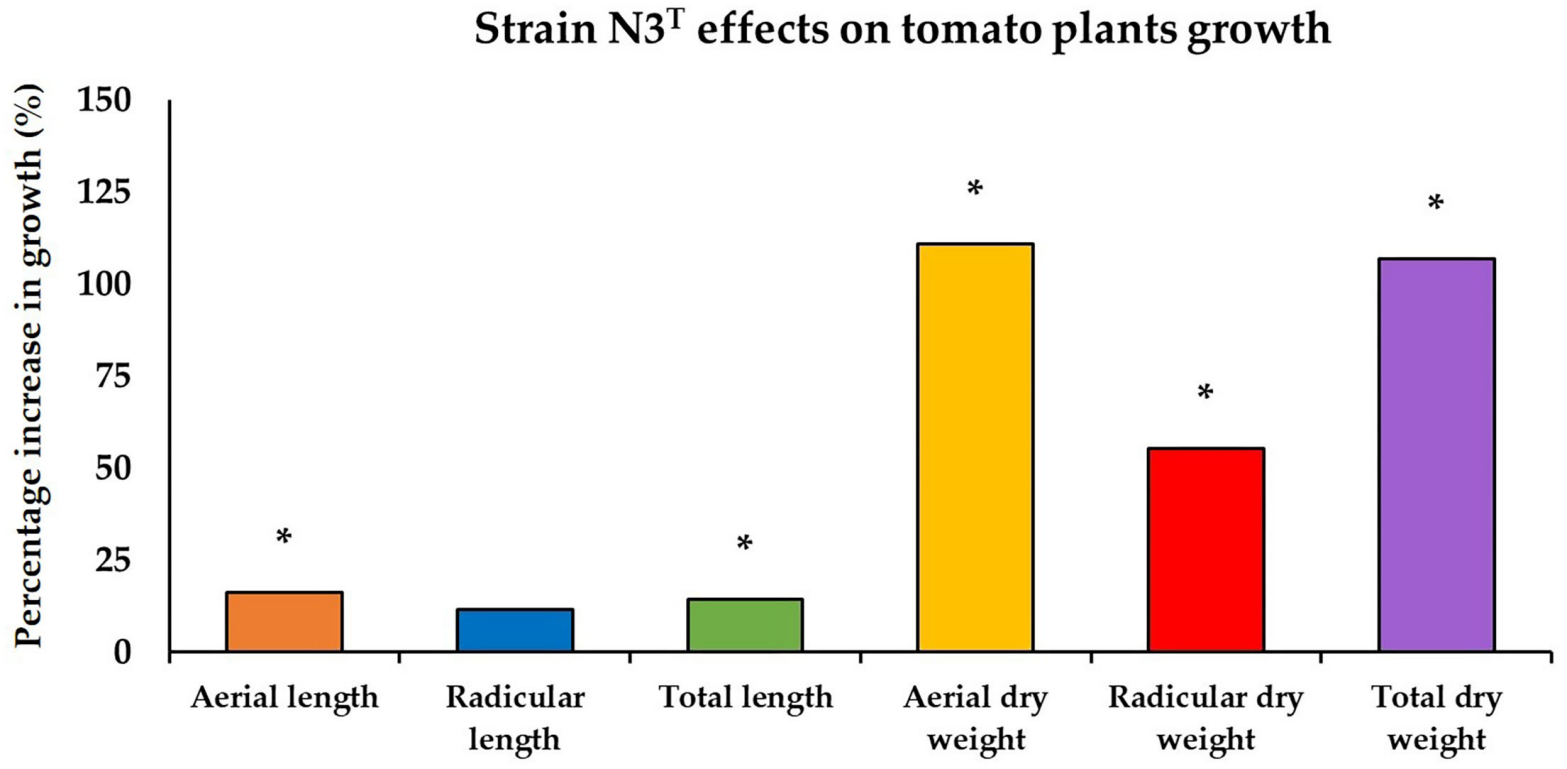
Plant growth-promoting activity of the strain N3^T^ in tomato plants with respect to control plants treated with water. *statistically significant difference (*p* < 0.01).

Experiments to test the PGP activity were performed with tomato because it is one of the most important vegetable plants in the world. Knowledge obtained from the studies conducted on tomatoes can be easily applied to these plants, which makes tomatoes an important research material (Kimura and Sinha, 2008). *In vivo* experiments to determine the growth promotion of the strain N3^T^ in tomato plants showed an increase in the length and dry weight of the plants compared with the control. Significant increases with respect to the control plants were observed in terms of aerial and total lengths, 16.1 and 14.3%, respectively, and of aerial, radicular, and total dry weight, 110.7, 55.3, and 106.8%, respectively (Figure 4).

The enzymatic degradation of AHLs in phytopathogens seems to be a promising alternative strategy to fight bacterial infections (Helman and Chernin, 2015). Previous studies reported the top 10 plant pathogenic bacteria in molecular plant pathology (Mansfield et al., 2012). The phytopathogens tested in this study, *D. solani* LMG 25993^T^ and *E. amylovora* CECT 222^T^ (members of the top 10), produce damages in potatoes and pears and cause huge losses in the agriculture production.

**Figure 5:**
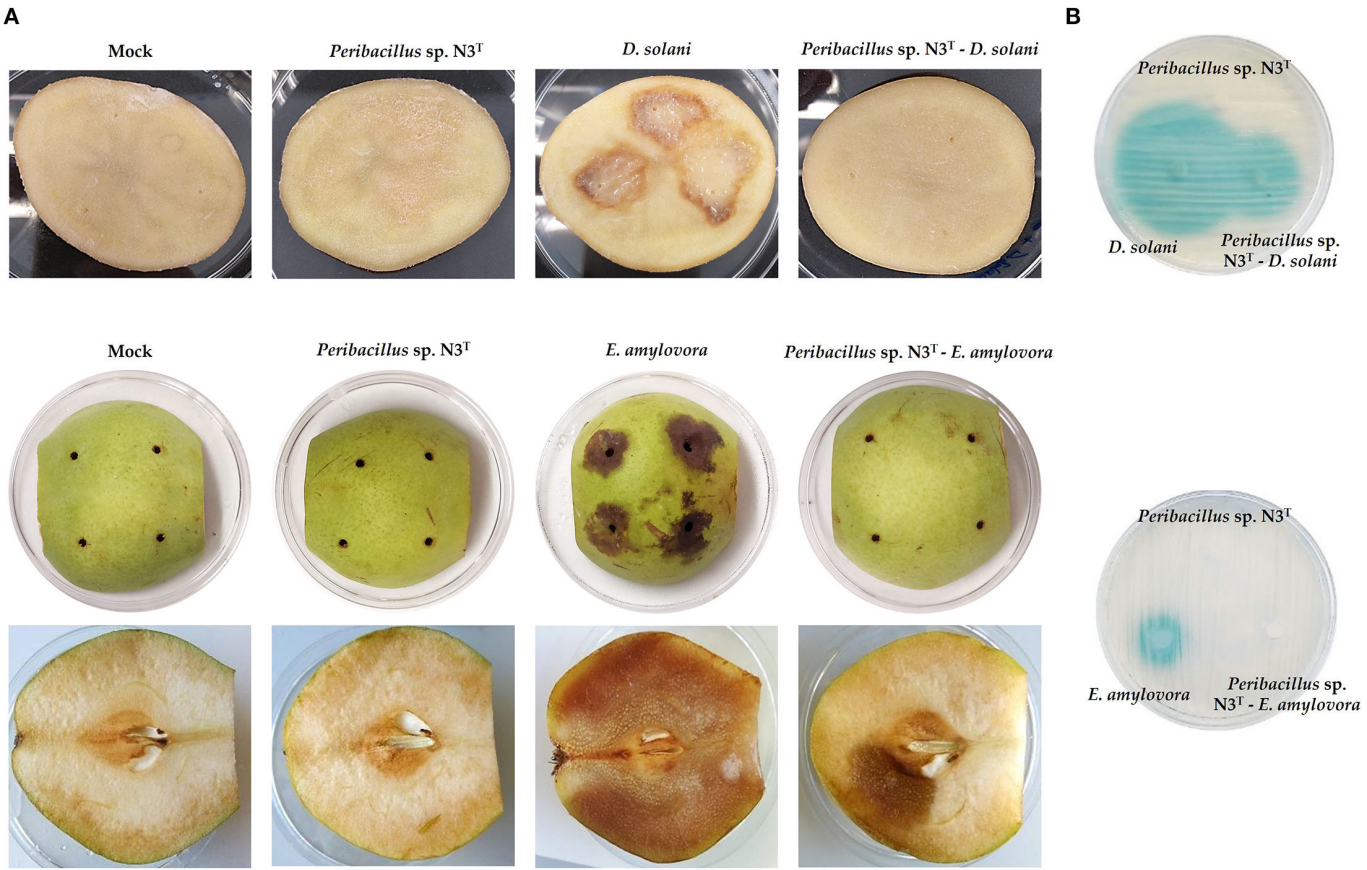
N3^T^ strain interference in *D. solani* and *E. amylovora* virulence on potato slices and pears **(A)**. Determination of N3^T^ quorum quenching activity on AHLs of *D. solani* and *E. amylovora* in AB-Xgal medium using *A. tumefaciens* NTL4 as biosensor **(B)**.

To evaluate the ability of the strain N3^T^ to interfere in the virulence of *D. solani* LMG 25993^T^ and *E. amylovora* CECT 222^T^, first, the QQ activity against AHL extracts from each pathogen strain was analyzed. A well-diffusion plate test showed a total degradation of the AHLs produced by *E. amylovora*, but a partial degradation was found on the *D. solani* AHLs extract when the N3^T^ strain was cocultured with them (Figure 5B).

Second, the cocultures of the strain N3^T^ and *D. solani* LMG 25993^T^ and *E. amylovora* CECT 222^T^ were carried out and tested for their virulence in potato and pear assay, respectively. Potato slices treated with *D. solani* LMG 25993^T^ in monoculture showed a tissue maceration of 26.3% (Supplementary Figure 3A), while those treated with the coculture of this pathogen and the strain N3^T^ showed a complete inhibition of this maceration (Figure 5A). In the case of the experiments carried out with *E. amylovora* CECT 222^T^ in pears, monoculture with *E. amylovora* CECT 222^T^ produced a 93.8% of tissue maceration in pears, and the percentage was reduced to 18.1% when pears were treated with the coculture of the strain N3^T^ (Supplementary Figure 3B). On the other hand, the N3^T^ strain did not cause any damage to the potato and pear tissues (Figure 5A). To discard the growth inhibition of the phytopathogens by the strain N3^T^, a plate-counting method was carried out using different concentrations of NaCl. The results indicated that no differences in the growth of *D. solani* and *E. amylovora* were observed in the monocultures with respect to the cocultures (10^7^ CFU mL^−1^).

These results indicated that the strain N3^T^ attenuated the virulence of the pathogens tested through a QQ approach. Our results are in line with other studies that also demonstrated the attenuation of phytopathogens virulence by using the QQ strains, such as *Lysinibacillus* sp. Gs50 (Garge and Nerurkar, 2016), *Stenotrophomonas maltophilia* M9-54 (Reina et al., 2019), *Pseudomonas segetis* P6 (Rodríguez et al., 2020), *Ochrobactrum intermedium* D-2 (Fan et al., 2020), and *Acinetobacter* sp. XN-10 (Zhang et al., 2020). In fact, heterologous expressions of QQ bacterial enzymes, such as the lactonases AiiA from *Bacillus* sp. or the metagenome derived HqiA (Torres et al., 2017) in *Pectobacterium carotovorum* subsp. *Carotovorum*, have demonstrated an effect on the production of pathogen virulence factors.

In many cases, the results shown in the laboratory conditions are not the same as the ones obtained under *in vivo* assays. Thus, in order to use the strain N3^T^ as an effective biocontrol agent in biotechnology, new experiments need to be done under greenhouse conditions. The preliminary results under *in vivo* assays suggest that the biocontrol mechanism of *Pseudomonas syringae* pv. *tomato* might be QQ *Peribacillus* sp. N3 (personal communication).

Other antivirulence mechanisms apart from QQ cannot be discarded in the strain N3^T^, since the *Bacillus* species are well-known to produce numerous inhibitor compounds and some of them do not affect the viability of the pathogen (Leathers et al., 2020). An analysis *in silico* using the antiSMASH tool (Blin et al., 2021) indicated that the strain N3^T^ produced lipopeptides of the family NRPS and lassopeptides that also implicated its antivirulence mechanism.

Although the *Bacillus* strains with growth promotion activity (Torres et al., 2019) or AHL-degrading enzymes from the *Bacillus* strains that can reduce the QS-regulated virulence factors in pathogens have been described (Dong et al., 2000; Zhao et al., 2008; Zhou et al., 2008), only a few studies have reported a bacterium that combines both properties (Vega et al., 2019; Rodríguez et al., 2020). Indeed, to our knowledge, this is the first study to describe a *Peribacillus* strain with both the PGP and QQ activities.

## Conclusion

The polyphasic taxonomic study as well as chemotaxonomic and genomic analyses showed that the strain N3^T^ isolated from river otter (*Lutra lutra)* feces constitutes a novel species (proposed name: *Peribacillus castrilensis* sp. nov.) within the genus *Peribacillus*, with the type strain N3^T^ (=CECT 30509^T^=LMG 32505^T^). Considering its plant growth promotion traits, the strain N3^T^ could constitute an alternative to increase the plant yield and thus reduce the inputs of chemical fertilizers in agriculture. Moreover, its ability to reduce the virulence factors' expression of *D. solani* and *E. amylovora* through QQ makes it able to be used as a biocontrol agent for fighting these pathogens, which is an alternative for antibiotic treatments currently used in the fields to fight them. Taken together, these results show the potential of this strain as a safe and eco-friendly alternative for agriculture to increase the field production and to reduce the economic losses.

## Description of *Peribacillus castrilensis* sp. nov.

*Peribacillus castrilensis* (cas'tri'len'sis. N.L. *castrilensis* for being isolated from Castril Natural Park, Granada, Spain).

*Peribacillus castrilensis* sp. nov. is motile, straight, Gram-positive, rod-shaped, which forms oval endospores in swollen sporangia. The colonies of *Peribacillus castrilensis* sp. nov. on TSA medium are cream colored after growing for 48 h at 28°C. This strain is halotolerant and can grow in the presence of 0.5–7.5% (w/v) NaCl concentrations, with 1% (w/v) as optimum. The cells grow in a temperature range of 10–42°C, with optimum growth at 28°C, and a pH range from 6 to 8, with pH 7 as optimum. *Peribacillus castrilensis* sp. nov. is a chemoorganotrophic and strictly aerobic microorganism. Under aerobic conditions, nitrate is not reduced, catalase is positive, and oxidase is negative. This strain is positive for arginine dihydrolase, tryptophan deaminase, and indole production but is negative for ONPG, lysine and ornithine decarboxylases, urease, citrate utilization, and acetoin and H_2_S production. Acids are not produced from any sugar tested except for aesculin, in which a weak reaction was detected.

The principal fatty acids of *Peribacillus castrilensis* sp. nov. are anteiso-C_15:0_ (68.07%) and iso-C_15:0_ (8.71%), and DNA G+C content was 40.3 mol% according to the *in silico* determination.

Type strain N3^T^ (= CECT 30509^T^ =LMG 32505^T^) was isolated from the feces of a river otter in Castril Natural Park in Granada (Spain). The GenBank/EMBL/DDBJ accession number for the 16S rRNA sequence of *Peribacillus castrilensis* N3^T^ is OL619301, and the complete genome is deposited under the accession number JAJNAF000000000.

## Data Availability Statement

The datasets presented in this study can be found in online repositories. The names of the repository/repositories and accession number(s) can be found in the article/Supplementary Material.

## Author Contributions

MR isolated the strain and performed the experiments. MR, JR, IL, IS, and FM-C conceived and supervised the study. IL, IS, and FM-C designed the experiments. JR performed the genomic analyses. MR, JR, and FM-C analyzed the data, prepared the figures, and wrote the manuscript. All authors have edited the manuscript and have agreed to the published version of the manuscript.

## Funding

This study was supported by a grant from the Spanish Ministerio de Educación y Ciencia (AGL-2015-68806-R), by a Grant from the Spanish Ministry of the Economy and Competitiveness (PID2019-106704RB-100/AEI/10.13039/501100011033), and from the Plan Andaluz de Investigación (Research Group BIO 188). MR was supported by a University of Granada Programme (Empleo Garantía Juvenil). JR was supported by an FPU fellowship from the Spanish Ministerio de Educación, Cultura y Deporte (FPU15/01717).

## Conflict of Interest

The authors declare that the research was conducted in the absence of any commercial or financial relationships that could be construed as a potential conflict of interest.

## Publisher's Note

All claims expressed in this article are solely those of the authors and do not necessarily represent those of their affiliated organizations, or those of the publisher, the editors and the reviewers. Any product that may be evaluated in this article, or claim that may be made by its manufacturer, is not guaranteed or endorsed by the publisher.
