# Supplementary material for: Peribacillus castrilensis sp. nov.: A Plant-Growth-Promoting and Biocontrol Species Isolated From a River Otter in Castril, Granada, Southern Spain
Source: Front Plant Sci. 2022 Jun 23;13:896728. doi: 10.3389/fpls.2022.896728 (PMC9262404; doi:10.3389/fpls.2022.896728)
Supplement: Supplementary file 1 [file Data_Sheet_1.docx]

## Supplementary Figures

**
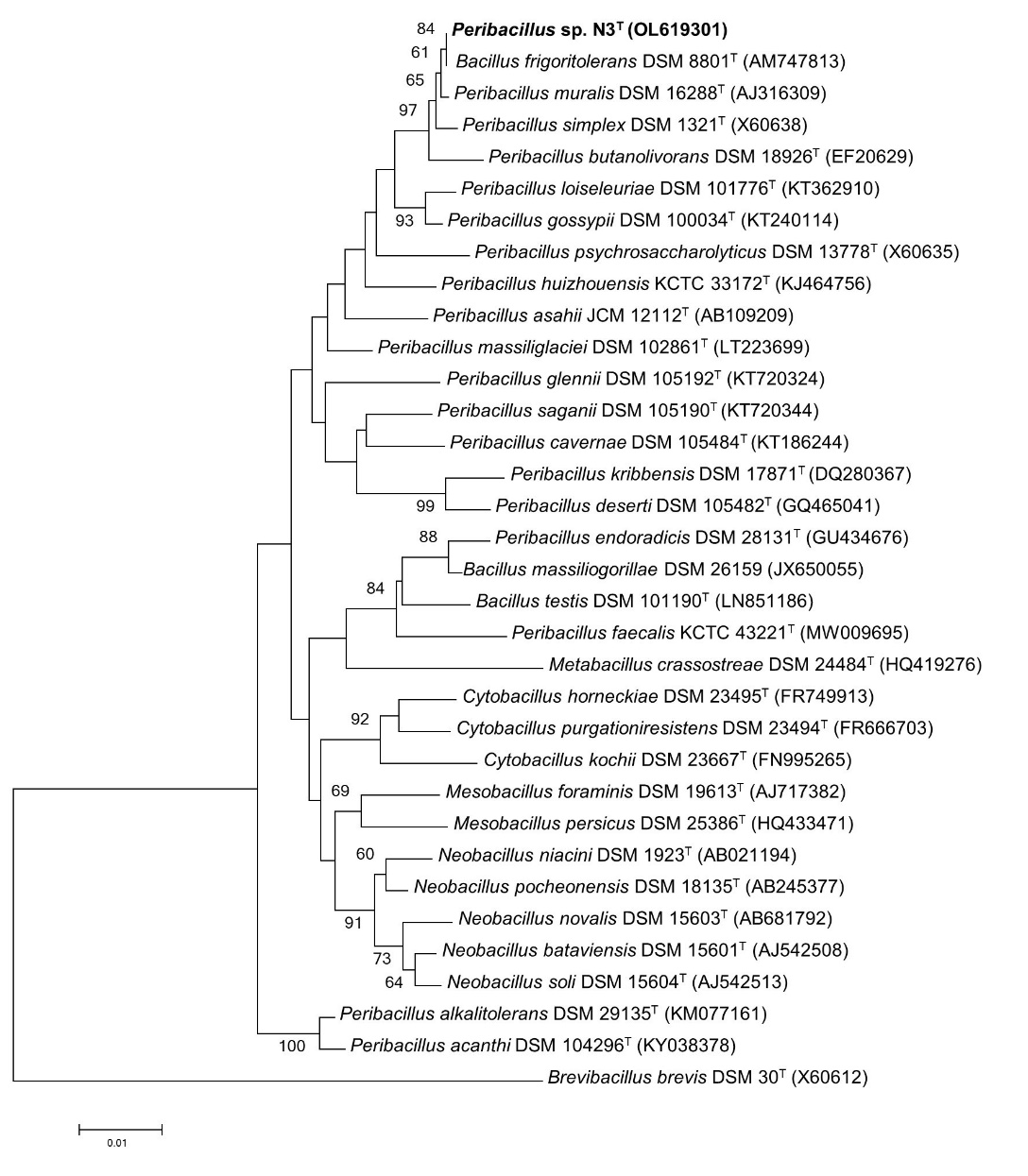
**

**Supplementary Figure 1**: Phylogenetic position of strain N3^T^ based on the neighbour-joining algorithm of the 16S rRNA gene sequence and its relationship with other related species. The GenBank/EMBL/DDBJ accession number of each sequence is shown in parenthesis. Bootstrap values are expressed as percentages of 1,000 replications, and those greater than 60% are shown at branch points. The evolutionary distances were computed using the Kimura 2-parameter method. The rate variation among sites was modelled with a gamma distribution (shape parameter = 1). Bar shows sequence divergence. Bar, 0.01 substitutions per nucleotide position. There were a total of 1238 positions in the final dataset. *Brevibacillus brevis* DSM 30^T^ sequence was used as the outgroup.


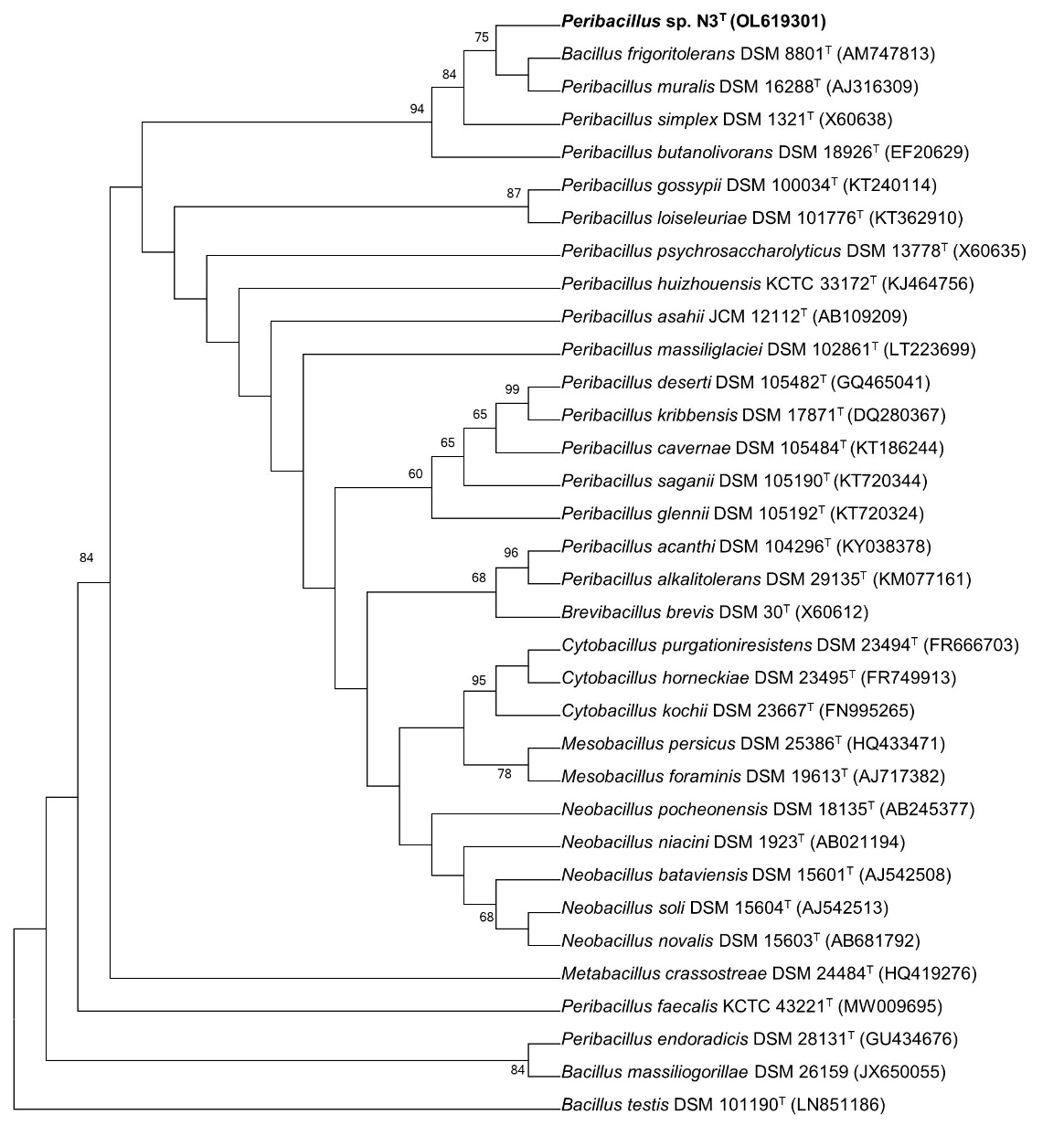


**Supplementary Figure 2**: Molecular phylogenetic analysis according to the maximum parsimony method. Evolutionary history was inferred by the aforementioned method using the Subtree-Pruning-Regrafting (SPR) algorithm. Bootstrap values are expressed as percentages of 1,000 replications, and those over 60% are shown at branch points. There were a total of 1238 positions in the final dataset. The Brevibacillus brevis DSM 30^T^ sequence was used as the outgroup.


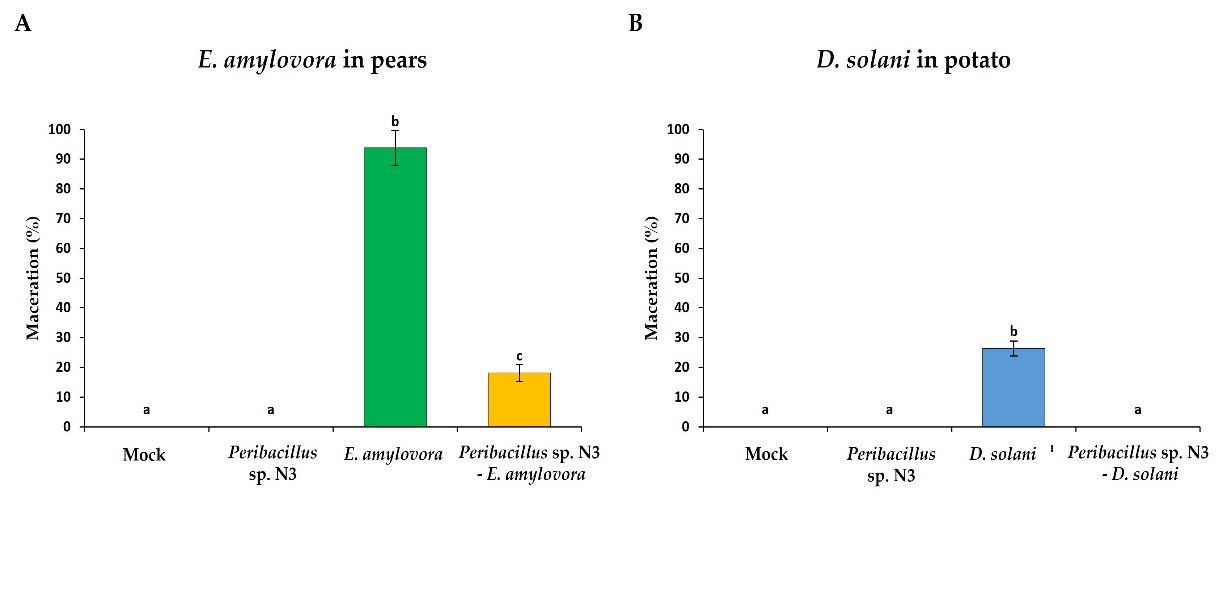


**Supplementary Figure 3**: Interference on E. amylovora (A) and D. solani (B) maceration in pears and potato through quorum quenching approach. Different letters indicate statistically significative differences (p < 0.01).

**Supplementary Table 1:** ANIb, ANIm (in brackets) and AAI (in parenthesis) values among the genomes of Peribacillus sp. N3^T^ (1) and its most related species: 2, P. simplex DSM 1321^T^; 3, P. butanolivorans DSM 18926^T^; 4, P. muralis DSM 16288^T^; 5, P. loiseleuriae DSM 101776^T^; 6, B. frigoritolerans DSM 8801^T^.

| **Strains** | **1** | **2** | **3** | **4** | **5** | **6** |
| --- | --- | --- | --- | --- | --- | --- |
| **1** | - | 92.96 [93.94]  (94.73) | 83.95 [86.61]  (87.42) | 83.55 [85.56]  (86.54) | 71.26 [85.32]  (67.54) | 67.72 [85.36]  (95.67) |
| **2** | 93.21 [93.94]  (94.73) | - | 84.93 [87.22]  (88.16) | 84.40 [86.83]  (86.96) | 71.73 [86.16]  (67.54) | 68.09 [85.01]  (94.43) |
| **3** | 84.28 [86.61]  (87.42) | 85.06 [87.22]  (88.16) | - | 81.52 [85.96]  (84.88) | 72.25 [85.96]  (68.81) | 67.59 [84.20]  (87.52) |
| **4** | 84.09 [86.57]  (86.54) | 84.48 [86.83]  (86.96) | 81.66 [85.33]  (84.88) | - | 70.47 [84.94]  (67.08) | 67.72 [85.19]  (86.56) |
| **5** | 71.46 [85.35]  (67.54) | 71.51 [86.00]  (67.54) | 72.58 [85.96]  (68.81) | 71.04 [84.93]  (67.08) | - | 67.98 [85.52]  (67.35) |
| **6** | 67.84 [85.32]  (95.67) | 67.78 [85.02]  (94.43) | 67.93 [84.21]  (87.52) | 68.02 [85.21]  (86.56) | 67.93 [85.51]  (67.35) | - |

**Supplementary Table 2:** Genome sequence similarity between *Peribacillus* sp. N3^T^ and genome sequences of its closely related type strains.

| **Strains** | **OrthoANI (%)** | **dDDH (%)** |
| --- | --- | --- |
| *P. simplex* DSM 1321^T^ | 93.66 | 69.10 |
| *P. butanolivorans* DSM 18926 ^T^ | 84.90 | 37.30 |
| *P. muralis* DSM 16288^T^ | 84.71 | 35.90 |
| *P. loiseleuriae* DSM 101776^T^ | 72.44 | 13.50 |
| *B. frigoritolerans* DSM 8801^T^ | 61.92 | 12.80 |

**Supplementary Table 3:** In vitro plant growth promoting traits of strain N3^T^. +, positive result; -, negative result; v, variable result.

| **Strain** | **Plant growth promotion trait** | | | | | | | | | | | | |
| --- | --- | --- | --- | --- | --- | --- | --- | --- | --- | --- | --- | --- | --- |
|  | **ACC deaminase** | **Acid phosphatase** | **Alkaline phosphatase** | **Caseinase** | **Cellulase** | **DNAse** | **Gelatinase** | **IAA production** | **Nitrogenase** | **Starch hydrolysis** | **Siderophores production** | **Tween 20 hydrolysis** | **Tween 80 hydrolysis** |
| N3^T^ | + | v | - | - | - | + | + | + | + | + | + | + | + |
